# Effect of 4 % chlorhexidine on cord colonization among hospital and community births in India: a randomized controlled study

**DOI:** 10.1186/s12887-016-0625-7

**Published:** 2016-08-02

**Authors:** Sushma Nangia, Usha Dhingra, Pratibha Dhingra, Arup Dutta, Venugopal P. Menon, Robert E. Black, Sunil Sazawal

**Affiliations:** 1 Department of Pediatrics, Lady Hardinge Medical College & Kalawati Saran Children’s Hospital, New Delhi, India; 2 Department of International Health, Johns Hopkins Bloomberg School of Public Health, 615 North Wolfe Street, Baltimore, MD 21205 USA; 3 Center for Public Health Kinetics, New Delhi, India; 4 Center for Micronutrient Research, Clinical Trials and Operational Research in Maternal and Child Health, Annamalai, Tamil Nadu, India

**Keywords:** Neonates, Newborns, Chlorhexidine, Colonization, Bacterial count, Cord cleaning, Umbilical cord, India

## Abstract

**Background:**

Infections are the single most important cause of neonatal mortality in developing countries. Results from trials in Asia evaluating the effect of chlorhexidine on neonatal mortality have been encouraging but limited data are available on the impact of cord cleansing on bacterial colonization. Further, no data from facility deliveries and impact with time is available. This pilot study was aimed to evaluate the impact of 4 % commercially prepared chlorhexidine on cord colonization and density of colonization among newborns in India.

**Methods:**

Three hundred twenty-six newborns (hospital-247; community-79) were enrolled within 24 h of birth and randomly assigned to one of three groups: chlorhexidine, placebo or dry cord care. Umbilical swabs were collected at baseline, 2- and 48- hours after intervention application.

**Results:**

At baseline, growth positivity (any bacterial growth) was 20 % (50 of 247 swabs) and 81 % (64 of 79 swabs) among hospital and community born neonates, respectively. In both settings, chlorhexidine compared to placebo and dry cord care, reduced colonization following 2- and 48-hour post application. Chlorhexidine significantly reduced 48-hour post application colony counts in comparison to placebo [Hospital: mean difference = −1.01; 95 % CI: −1.72, −0.30 Community: mean difference = −1.76; 95 % CI: −2.60, −0.93] and dry cord care [Hospital: mean difference = −1.16; 95 % CI: −1.93, −0.39 Community: mean difference = −2.23; 95 % CI: −3.18, −1.29]. Differences were similar for gram-positive and gram-negative bacteria.

**Conclusions:**

Cord cleansing with 4 % chlorhexidine soon after birth reduced colonization as well as density of colonization significantly; however this pilot study does not address the impact of chlorhexidine on mortality. The control preparation neither increased or decreased colonization.

**Trial registration:**

Clinical Trial Registration: clinicaltrials.gov: NCT01528852, Registered February 7, 2012.

**Electronic supplementary material:**

The online version of this article (doi:10.1186/s12887-016-0625-7) contains supplementary material, which is available to authorized users.

## Background

Neonatal deaths account for 40 % of global under-five mortality [1–3] and are a major health concern in the developing countries, particularly in the south Asian and sub-Saharan countries [4, 5]. Each year serious infections account for nearly 13 % of the 3 million neonatal deaths, this proportion is closer to 50 % in settings with high mortality risk [2]. Five countries namely India, Nigeria, Democratic Republic of the Congo, Pakistan and China alone account for half (2.440 million) of the global deaths from infections and 53.3 % (1.636 million) of neonatal deaths [5]. Community based studies from developing world suggest that infections are responsible for 8 to 80 % of all neonatal deaths and as many as 42 % deaths in first week of life [5, 6].

In neonates, umbilicus acts as a bacterial reservoir and a potential entry point for the infection, especially in first few days of life, when umbilical vein is patent. This may lead to sepsis with or without omphalitis [7, 8]. The responsible organisms most likely originate in maternal genital tract and are acquired during labor and delivery [9]. In low income countries, many neonatal infections are environmentally acquired because of higher number of home deliveries, unsafe traditional practices, untrained birth attendants and unclean living conditions, all of which pose an increased risk of umbilical cord infection [10]. The local signs of umbilical cord infection include pus, redness, swelling, warmth, tenderness and foul odour [11] and seem to be associated with increased risk of mortality [12]. Infectious organisms may get directly transmitted from patent umbilical cord to the blood stream without evident signs of local cord infection [13].

Recommendations for umbilical cord care, particularly in regard to prevention of umbilical cord infection, are controversial. In developing countries, World Health Organization (WHO) [10, 14] promotes dry cord care. These recommendations were based on lack of evidence for alternative approaches [14]. Based on currently available evidence, the WHO recommends 7.1 % chlorhexidine digluconate solution or gel, (delivering 4 % chlorhexidine) among home deliveries in settings with neonatal mortality rate more than 30 [15]. This interim recommendation awaits review of further data especially the results of two large trials in Africa.

Of topical antiseptics (eg, ethanol, silver sulfadiazine, triple dye, gentian violet, chlorhexidine, povidine iodine), chlorhexidine with strong residual activity has shown potential as an effective cord care agent during the neonatal period against both gram-positive and gram-negative organisms [16–18]. It has an excellent safety profile, is rarely associated with bacterial resistance, is easy to administer and costs few cents per application [9, 19, 20]. Available high-quality evidence from the recently conducted Cochrane review indicates that cord cleansing with 4 % chlorhexidine reduces the risk of neonatal mortality by 12 % and sepsis (omphalitis)/infections by 50 % in low-resource community settings including Nepal, Bangladesh, and Pakistan [21]. However, in hospital settings, chlorhexidine cord cleansing reduces the risk of omphalitis/infections by 52 % and may lead to no difference in neonatal mortality as compared to dry cord care. Despite all these data, limited evidence is available on the mechanism of this protective effect [22–24] and only one study [25] published recently suggested an impact on bacterial colonization of the cord and provided information on early neonatal colonization dynamics in community setting. There are no data available evaluating impact of chlorhexidine on colonization among facility births, and spectrum of chlorhexidine overtime. In all the earlier chlorhexidine trials evaluating impact on mortality and/or cord infections, 4.0 % free chlorhexidine were prepared by diluting 20 % chlorhexidine digluconate to the appropriate concentration with purified water at the study sites. As there was no data available from Africa, two large trials in Africa (Pemba, Tanzania and Zambia) were funded by Bill and Melinda gates Foundation to evaluate the mortality impact of the intervention. In the present pilot study, we tested the antimicrobial activity of the commercially prepared 4 % chlorhexidine solution as a cord-cleansing agent, and evaluated its impact on bacterial colonization and colony counts after 2- and 48-hour post-application in hospital- and community born neonates in comparison to placebo (same solution without active ingredients) and dry cord care. The aim of this pilot study was to ensure and document the efficacy of preparation before evaluating mortality and sepsis impact in large trials (more than 60,000 newborns in African setting). In addition, the pilot study provided data on potential cord colonizing pathogens that could be responsible for sepsis, apart from regular skin flora.

## Methods

### Study design

This pilot study was a prospective, randomized, controlled trial conducted in New Delhi, India. There could be significant differences in environmental cleanliness and bacteriologic profile in the community and hospital settings; study subjects from both the settings were included in order to have a representative sample.

### Study population

The hospital component of the pilot study was conducted at Kalawati Saran Children’s Hospital, New Delhi while the community component was undertaken at Sangam Vihar, New Delhi.

Kalawati Saran Children’s Hospital is a government hospital catering to patients from the lower socio-economic strata. Approximately 40–60 deliveries take place daily in this hospital.

Sangam Vihar is a resettlement colony mainly comprising of migrants from rural areas of the country. Its population is predominantly from the low socio-economic strata of the society. Almost 60 % of the births in Sangam Vihar take place at home (20–25 deliveries daily).

### Training and reliability

Training sessions were organized for the field workers, supervisors and hospital staff who were apprised of the study protocol and the various forms that need to be filled at different time points. Swab sample collection from the umbilical cord as well as cleaning of cord using the assigned intervention method was demonstrated using a dummy doll. This was followed by practice and return demonstration by trainees on the dummy. Subsequently, dry run sessions were undertaken by the staff to ensure reliability and effective implementation of study protocol.

### Recruitment and enrollment

#### Enrollment in hospital

Study team was stationed outside the maternity ward from 8.00 am to 5.30 pm. After birth, the study team comprising of hospital staff and study supervisor visited the mother baby duo and screened the newborn for eligibility to participate in the study. If the newborn was found eligible (normal delivery, full term healthy newborns of both sexes with birth weight >2500 g, first contact ≤48 h), the study purpose and procedure were explained to the mother once she was stable or else to the nearest kin and the consent for their newborn’s participation in the study was sought. The newborn was enrolled in the study after the consent was obtained from the parents. Neonates requiring resuscitation and admission to NICU and also those with major congenital malformation were excluded.

#### Enrollment in community

A survey was carried out by trained birth attendants (TBAs) to identify the pregnant women in the six blocks of Sangam Vihar. A record of all identified pregnancies with their tentative due delivery dates was prepared. The family members and TBAs were instructed to contact the study supervisor at the time of delivery or immediately thereafter. The study supervisor and fieldworker along with the TBA visited the newborn within 48 h of delivery. They screened the newborn for eligibility (vaginally delivered, term healthy newborns, first contact ≤48 h) and if found eligible, the study purpose and procedure were explained to the mother and the consent for their newborn’s participation in the study was sought.

### Sample size estimations

Based on Nepal hospital data (Hodgins et al [26]) the sample size was estimated to be 80 in each intervention group for the hospital setting, with an alpha of 0.05 and power of 80 %, to detect a reduction in bacterial colonization from 29 to 11 % in the chlorhexidine group. Based on Bangladesh study (Mullany et al, personal communication), the sample size was estimated to be 44 in each intervention group in the community setting, with an alpha of 0.05 and power of 80 %, to detect a reduction in bacterial colonization from 93 to 69 % in the chlorhexidine group.

### Masking and randomization

All enrolled neonates were randomly allocated to 1 of the 3 intervention groups: (a) 4 % chlorhexidine or (b) placebo (mild soap water) or (c) dry cord care group. Both the chlorhexidine and the placebo groups were blinded and the preparations were identical in packaging, appearance, colour, consistency and odour. It was not possible to blind the allocation to the dry cord care group. Each intervention group was identified by a letter code. The manager in the manufacturing unit decided the codes to be used for the solutions and kept the information secret till the data analysis was complete. Thus, neither the mothers of the neonates nor the hospital/field staff knew which intervention was being used. Two separate randomization lists were prepared; one for the hospital and other for the community births. For the hospital, a randomization list containing running serial number and randomly allocated code (A through I) was generated. These letter codes were used to identify three intervention groups. In order to ensure equalization of groups, six letter codes for the solution (3 codes for chlorhexidine and 3 for placebo groups) and three letter codes for the dry cord care were used. In-house computer software generated a random sequence of group codes with permuted block length of 18. Neonates were allocated to one of the groups in the order in which they got enrolled. For the community births, similar randomization procedure was used.

### Intervention description

Intervention was prepared by Galentic Pharma (India) Pvt. Ltd, Mumbai, India. Table 1 shows the composition of the chlorhexidine and placebo solution. For the intervention stability, potency, colour, odour and consistency, the preparations were tested at various stages.

**Table 1:** Composition of chlorhexidine and chlorhexidine placebo

Ingredients	Chlorhexidine 4 % solution (% *v*/*v*)	Placebo solution (% *v*/*v*)
Chlorhexidine gluconate 20 % *w*/*v* solution BP	35.70	-
Polyoxyl 40 hydrogenated castor oil NF (RH 40)	0.80	0.80
Carmoisine	0.0005	0.0005
Purified water BP	Q.S to 100.00	Q.S to 100.00
Isopropyl alcohol BP	4.00	-
Mild soap [Sodium lauryl sulphate^a^ (STEROCARE SLS (L))]	-	0.40
Silicon antifoaming agent^a^	-	0.001

Note: ^a^sodium lauryl sulfate is used to match the foaming which is seen in chlorhexidine 4 % solution and silicon antifoaming agent is used to avoid the excess foam which forms in the placebo

### Study procedures

#### Baseline (0 h) swab collection

For hospital births, soon after delivery, hospital supervisor checked the hospital records to record birth related information. Before beginning the procedures, the resident doctor washed his/her hands and donned sterile gloves. The resident doctor on duty collected the swab using HIMEDIA HiCulture^TM^ transport swabs with amies medium with charcoal (Cat no: MS 651) from the tip, stump and base of the umbilicus and peri-umbilical region (2.5 cm radius around umbilicus) and the hospital supervisor immediately put the bar-coded sticker on the swab tube and pasted the corresponding duplicate sticker on the form. The date and time of the collection of each swab was recorded on a form. Within 4 h of collection, the swabs were transferred to the laboratory for culture analysis. Field/study worker used the same procedure to collect swabs among the community births.

#### Application of intervention (immediately following the collection of swab)

After the collection of swab for culture analysis, hospital staff/fieldworker applied the assigned intervention immediately. She/he opened the designated intervention pack and applied the intervention to cleanse the tip, stump and base of the umbilicus and peri-umbilical region. The intervention was applied twice: first after the collection of baseline swab and second 24 h after first application. In dry cord group, no intervention was applied and the standard WHO guidelines for cord care were followed [10].

#### Swab collection after 2-hours and 48-hours of application of intervention

In both settings, 2-hours and 48-hours post application, the study staff collected the umbilical swab, following the same procedure used for pre-application swab collection. In case of dry cord, the sample collection and timing was matched i.e. it was taken 2- and 48- h after the first sample.

#### Culture analysis

The culture analysis for umbilical cord swabs was performed at Dr Dang’s Medical Diagnostic Center, Hauz Khas, New Delhi, India. Umbilical swabs collected using HiCulture^TM^ Transport Swabs (Cat No. MS651) were transported to laboratory for aerobic culture. All samples (i.e. 10 μL of the neat specimen) were inoculated in 3 media plates i.e. McConkey Agar, Blood Agar and Chocolate Agar. The plates were incubated as per recommended time and temperature and were checked for absence or presence of bacterial isolates. Smear was prepared for identifying gram-negative and gram-positive bacteria. Based on the morphology and colour of the stain taken by the bacteria, they were classified into gram-positive or gram-negative organisms. Samples were further tested for species identification using standard manual methods and Vitek 2 compact system from BioMerieux France. For colony counts, the samples were put in 1 ml of normal saline for 3 log dilution and sub-cultured using standard subculture technique [27]. Presence of bacterial growth, identification to gram-positive/-negative bacterial organisms and semi-quantitative colony count was estimated for all samples. Laboratory staff members were blinded to the formulation used; the swabs and the accompanying forms were labeled with bar-coded stickers. In addition, various quality control measures were followed using standard American Type Culture Collection strains during each step and inter/intra observer reliability tests in microscopy.

#### Statistical analysis

We used Visual Basic 6.0/ASP.net and Oracle 8i to manage the data, with stringent range, consistency, and logical checks. To ensure data quality and accuracy, real time data entry was done using netbook. Data were analyzed separately for the hospital and community births using Statistical Package of Social Sciences (SPSS Inc., Chicago, Illinois, USA, SPSS, Version 19.0 for Windows) and Stata (Stata Corp., College Station, Texas, USA, Intercooled Stata 12.0 Version), *P* ≤ 0.05 was considered statistically significant for all analyses. Descriptive statistics (frequencies, percentages, means and standard deviation) were calculated. We examined the characteristics of newborns and mothers across the allocated groups on a range of variables to determine the degree of balance achieved by the randomization. We analyzed colonization positivity data by intervention groups and follow-up time. Among those that were positive, we further estimated distribution of colony counts by intervention groups and follow-up time. A paired analysis comparing the baseline positivity/colony count of the child with his 2- and 48-hours post intervention positivity/counts was performed to study any group differences as well as changes over follow-up time within group. The chi square, *t-* test and OR with 95 % CI were used to estimate statistics and significance.

#### Outcomes

The primary outcome was culture positivity at 2-hours and 48-hours post application and the secondary outcome was bacterial colony count from umbilical and peri-umbilical region to estimate the concentration of viable bacteria. The reduction in bacterial colonization was assessed as proportion of neonates with positive culture (from umbilical and peri-umbilical area) 2-hours and 48-hours after application of 4 % chlorhexidine solution as compared to dry cord care and control groups. The reduction in density of bacterial colonization (limited to samples with growth) after 2-hours and 48-hours of application of 4 % chlorhexidine solution as compared to dry cord care and control groups was assessed in terms of mean reduction in bacterial colony counts. The risk of colonization stratified by gram-positive and gram-negative organisms was estimated by intervention groups.

## Results

### Participants

**Fig. 1:**
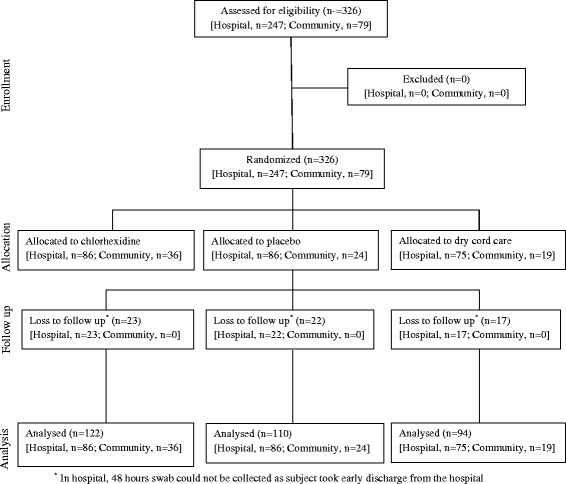
Study participants flow diagram

Between November 6, 2010 and December 10, 2010 and then from January 31, 2011 to February 9, 2011, a total of 326 newborns both from the hospital (n-247) and community (n-79) were enrolled in the study. In both the settings, newborns were randomly assigned to chlorhexidine, placebo or dry cord care group and umbilical swabs were collected at baseline (before the application of intervention), 2-hour and 48-hour after the application of the assigned intervention (Fig. 1). Of the 247 neonates enrolled from the hospital, 48-hour swab sample could not be collected in 62 neonates (chlorhexidine-23, placebo-22, dry cord care-17) as the subjects took early discharge from the hospital and only contributed the first (baseline) and second (2-hour) swabs. The rate of discharge before the third swab did not differ between the 3 intervention groups. A total of 916 umbilical swabs (hospital 679, community 237) were collected. There was no reported adverse event in the intervention groups in both the settings.

### Baseline characteristics

#### Hospital data

In hospital, among the 247 enrolled neonates, 86 were allocated to chlorhexidine, 86 to placebo, and 75 to dry cord care. Comparison of baseline characteristics is shown in Table 2. All groups were comparable as regards the baseline characteristics such as age of the mother, mode of delivery, and birth weight of newborns etc. The proportion of males was higher in groups other than the chlorhexidine group. Risk factors for infection such as prolonged labor >24 h, leaking per vagina >24 h, meconium staining, and maternal urinary tract infection were present but the proportion was very low. The time since birth when the neonate got enrolled in the study was similar across the three groups.

**Table 2:** Baseline characteristics and risk factors for infection^a^

Variables	Chlorhexidine	Placebo	Dry cord
Hospital data	(*n* = 86)	(*n* = 86)	(*n* = 75)
Age of mother (years, mean ± SD)	23.87 ± 2.54	23.77 ± 2.86	25.12 ± 3.24
Vaginal delivery	100.0	100.0	100.0
Male births	48.8	64.0	58.7
Birth weight (grams, mean ± SD)	2728.3 ± 479.5	2684.3 ± 453.6	2794.8 ± 437.6
No. of pelvic examinations (mean ± SD)	2.37 ± 1.25	2.07 ± 1.00	2.00 ± 0.97
Problems during delivery			
Meconium staining	2.3	2.3	5.3
Prolonged labor	2.3	1.2	1.3
Maternal risk factors for infection			
LPV^b^ > 24 h	4.7	2.3	2.7
Chorioamnionitis	0.0	0.0	0.0
Fever in mother	1.2	0	1.3
Maternal UTI	0.0	0.0	1.3
Time since birth (hrs:min, mean ± SD)	4:39 ± 2.31	4:35 ± 2:49	4:12 ± 2:37
Community data	(*n* = 36)	(*n* = 24)	(*n* = 19)
Age of mother (years, mean ± SD)	24.42 ± 3.95	24.62 ± 4.13	25.79 ± 3.91
Male births	38.9	50.0	52.6
Did the person clean hands before conducting delivery	97.2	100.0	100.0
New razor blade for cutting the cord	100.0	95.8	100.0
Baby given bath immediately after delivery	94.4	100.0	100.0

^a^Values are in percentages unless specified

^b^ *LPV* leaking per vagina

#### Community data

Of the 79 neonates enrolled in the community; 36 were assigned to chlorhexidine, 24 to placebo and 19 to dry cord care groups. At baseline, no significant differences were seen in a variety of variables measured, such as age of the mother, cord cutting methods, hand cleaning before conducting delivery and babies receiving bath immediately after delivery. The proportion of males was lower in chlorhexidine group (Table 2).

### Bacterial colonization and density of colonization among positive cultures

#### Hospital data

At baseline, proportion of positive swabs was 20 % (50 of 247 swabs) among hospital born neonates; it was little higher in chlorhexidine group (26.7 %) than dry cord care (18.7 %) and placebo (15.1 %) groups. Chlorhexidine group showed reduction in colonization in both 2-hours and 48-hours post application in comparison to baseline. However, there was an increase in growth positivity rates following 2-hour and 48-hour post application in dry cord (91.4 %) and placebo groups (90.6 %). Among those with positive culture results, colony counts were observed to assess if there was any intervention impact on density. In the chlorhexidine group, compared to placebo and dry cord care group, the mean colony count at the 2-hour and 48-hour follow-up was lower than the baseline (Table 3) indicating that chlorhexidine was effective in reducing the bacterial load.

**Table 3:** Bacterial colonization (proportion with positive culture) and colony counts by intervention group and time of swab collection

Variables	Baseline	2-hour post intervention	48-hour post intervention
Hospital data			
Bacterial colonization^a,b^			
Chlorhexidine (n-86, 86, 63)	23 (26.7)	8 (9.3)	12 (19.0)
Placebo (n-86, 86, 64)	13 (15.1)	23 (26.7)	58 (90.6)
Dry Cord (n-75, 75, 58)	14 (18.7)	24 (32.0)	53 (91.4)
Colony counts (Limited to samples with growth)^c,d^			
Chlorhexidine (n-23,8,12)	3.67 ± 1.15	3.01 ± 0.88	3.96 ± 1.69
Placebo (n-13,23,58)	3.71 ± 0.75	3.77 ± 1.06	4.97 ± 0.97
Dry Cord (n-14, 24, 53)	3.88 ± 0.91	3.96 ± 1.69	5.12 ± 1.07
Community Data			
Bacterial colonization^a,b^			
Chlorhexidine (n-36, 36, 36)	30 (83.3)	16 (44.4)	14 (38.9)
Placebo (n-24, 24, 24)	20 (83.3)	15 (62.5)	16 (80.0)
Dry Cord (n-19, 19, 19)	14 (73.7)	14 (73.7)	16 (84.2)
Colony Counts (Limited to samples with growth)^c,d^			
Chlorhexidine (n-30, 16, 14)	5.28 ± 1.00	3.85 ± 1.15	3.32 ± 1.36
Placebo (n-20, 15, 16)	5.25 ± 1.10	5.35 ± 0.77	5.08 ± 0.80
Dry Cord (n-14, 14, 16)	5.85 ± 1.31	5.76 ± 1.24	5.55 ± 0.93

^a^Bacterial colonization was defined as the growth of any organism from the sample; each swab was defined as positive or negative. The total proportion of neonates positive for any organism was estimated at specific time points i.e. at baseline, 2 and 48 h post intervention and was compared across 3 intervention groups

^b^Values are expressed in N (%)

^c^Colony counts were estimated among the positive culture swabs at baseline, 2-hour and 48- hour post intervention in three intervention groups to measure the density of bacterial colonization. The counts were measured in terms of colony-forming units (CFUs) per ml

^d^Values are expressed in mean ± SD

Table 4 presents the paired comparison between the groups for bacterial colonization and colony counts. Comparison of change in growth positivity from baseline to 2-hours post application showed 80 % reduction with chlorhexidine application in comparison to placebo [OR = 0.20; *p* = 0.001] and 81 % reduction compared to dry cord care [OR = 0.19; *p* < 0.001]. There was a 98 % reduction in change in growth positivity between baseline and 48-hour post application [chlorhexidine vs. placebo: OR = 0.02; *p* < 0.001 and chlorhexidine vs. dry cord: OR = 0.02; *p* < 0.001]. Chlorhexidine showed significant reduction in change in colony counts from baseline to 48-hour post application in comparison to placebo [difference in mean = −1.01; *p* = 0.006] and dry cord [difference in mean = −1.16; *p* = 0.004].

**Table 4:** Comparison between chlorhexidine vs. placebo/dry cord care for the bacterial colonization and colony counts

Variables	Chlorhexidine vs. placebo		Chlorhexidine vs. dry cord	
	Odds ratio (95 % CI)	*p* value	Odds ratio (95 % CI)	*p* value
Hospital				
Paired comparison for bacterial colonization between-				
Baseline and 2-hour post intervention	0.20 (0.08–0.52)	0.001	0.19 (0.08–0.47)	<0.001
Baseline and 48-hour post intervention	0.02 (0.008–0.07)	<0.001	0.02 (0.007–0.07)	<0.001
Difference in mean of colony counts (95 % CI) at 48 h	−1.01 ( −1.72– −0.30)	0.006	−1.16 ( −1.93– −0.39)	0.004
Community				
Paired comparison for bacterial colonization between-				
Baseline and 2-hour post intervention	0.47 (0.16–1.37)	0.17	0.14 (0.03–0.71)	0.02
Baseline and 48-hour post intervention	0.17 (0.05–0.63)	0.008	0.10 (0.02–0.51)	0.006
Difference in mean of colony counts (95 % CI) at 48 h	−1.76 ( −2.60– −0.93)	<0.001	−2.23 ( −3.18– −1.29)	<0.001

#### Community data

Among community births, baseline positivity was 81 % (64 of 79 swabs) (Table 3). Chlorhexidine showed a non-significant reduction of 53 % (OR = 0.47; *p* = 0.17) and a significant reduction of 86 % (OR = 0.14; *p* = 0.02) in change in bacterial colonization from baseline to 2 h sample as compared to placebo and dry cord care groups, respectively (Table 4). There was also a significant 83 % reduction in change in bacterial colonization from baseline to 48-hour post application in chlorhexidine group compared to placebo; 90 % compared to dry cord care. Change in mean colony counts from baseline to 48-hour post intervention among growth positives in chlorhexidine group were significantly reduced in comparison to placebo [difference in mean = −1.76; *p* < 0.001] and dry cord care [difference in mean = −2.23; *p* < 0.001].

### Specific organisms identified in the hospital and community setting

Among swabs with positive culture, the presence of specific organisms was assessed. In the hospital setting, *Acinetobacter* spp., *Citrobacter diversus*, *Citrobacter* spp., Coagulase-negative *Staphylococcus*, *Escherichia coli*, *Klebsiella* spp., *Pseudomonas aeruginosa*, *Pseudomonas* spp., *Staphylococcus aureus*, *Staphylococcus* spp. and *Viridans streptococci* were the most common organisms, constituting both gram-positive and gram-negative strains, identified on the umbilical stump, base and the peri-umbilical region. However, in the community, *Acinetobacter iwoffii., Acinetobacter junii*, *Acinetobacter baumanii*, *Acinetobacter haemolyticus*, *Acinetobacter* spp., *Acinetobacter ursingii, Aeromonas hydrophilia*, *Aeromonas spp.*, *Cedecea davisae*, *Citrobacter diversus*, *Citrobacter* spp., Coagulase Negative *Staphylococcus*, *Escherichia Coli*, *Enterobacter cloacae*, *Klebsiella pneumoniae*, *Klebsiella* spp., *Pseudomonas aeruginosa*, *Staphylococcus aureus*, *Staphylococcus lentus*, and *Staphylococcus sciuri* were the most common pathogens identified on the umbilical cord.

When colonization positivity data was analyzed by gram-positive/gram-negative strains, intervention groups and follow-up time, the reductions with chlorhexidine persisted. In hospital born neonates, the overall reduction in colonization for gram-positive as well as gram-negative organisms remained significant for the chlorhexidine group compared to placebo or dry cord care groups for 2-hours and 48-hours post application samples. For the community births, there was a significant reduction in the chlorhexidine group compared to placebo and dry cord for gram-positive bacteria. For the gram-negative bacteria, although there was a trend of reduction, the differences were significant only at 48-hour post application (Additional file 1).

## Discussion

Chlorhexidine application over umbilical cord has been shown to reduce the risk of omphalitis and neonatal mortality in community settings in recent trials done in Nepal, Pakistan and Bangladesh [21, 28]. In this double blind randomized controlled trial with facility and community data, we evaluated the impact of 4 % chlorhexidine cord cleansing compared to placebo or dry cord care on cord colonization. Cord cleansing with 4 % chlorhexidine showed significant reduction in colonization and density of pathogens in both facility and community setting. As compared to dry cord care, the impact of 4 % chlorhexidine was greater in the first 48 h after birth suggesting that chlorhexidine may be a possible intervention for reducing omphalitis and sepsis arising from transmission of bacterial infections via the umbilical remnant. These findings are in line with observations reported in a study conducted in Bangladesh [29, 30].

The potential mechanisms for the chlorhexidine action in reducing the colonization rates could possibly be the result of the increased binding efficiency of the detergent form to the umbilical tissues, resulting in prolonged residual effect [30]. This bactericidal effect of chlorhexidine could be attributed to its chemical structure, which is a positively charged hydrophobic and lipophilic molecule that interacts with phospholipids and lipopolysaccharides on the cell membrane of bacteria and enters the cell either through active or passive transport mechanism [31]. Interaction of the positive charge of the molecule with the negatively charged phosphate groups on microbial cell wall [32, 33] alters the cells’ osmotic equilibrium, increasing the permeability of the cell wall thus allowing the chlorhexidine molecule to penetrate into the bacteria [34]. Damage to this delicate membrane is followed by leakage of intracellular constituents, particularly phosphate entities such as adenosine triphosphate and nucleic acids. As a consequence, the cytoplasm becomes congealed, with resultant reduction in leakage; thus, there is a biphasic effect on membrane permeability. These early changes are beneficial during the critical first few hours and days of life when most neonatal deaths occur in resource-poor settings [4]. Our data also suggests that mild soap and water solution (placebo) was not very effective in reducing bacterial colonization in comparison to dry cord care. This placebo solution only reduced the pathogens in immediate 2-hours post application swabs in the community setting and did not produce extended effect for 48 h. This could be a mere chance finding or cord cleansing process itself mechanically removed the organisms from the umbilical and peri-umbilical region, subsequently decreasing the bacterial load. This indicates that for residual and cumulative effects, antibacterial agents such as chlorhexidine are substantially more effective than non-antiseptic agents [20].

Bacteriologic colonization rates, profile, and dynamics in a community setting might differ substantially from that in the hospital setting [35]. In the present study, the observed colonization positivity rate was higher in the community-setting (81 %) than in the hospital (20 %) which could be due to unhygienic environment, unclean delivery practices at homes and immediate post-delivery traditional practices. Further, as compared to other studies, the overall pre-application bacterial growth was low in the hospital setting which could be due to differences in neonate handling and infection prevention practices [26, 35, 36]. In the present study, the reported impact of 4 % chlorhexidine on the reduction of bacterial colonization was consistently high in both settings.

This study also describes the specific pathogens colonizing the umbilical cord. Trends in colonization positivity data were assessed by intervention groups, follow-up time and gram stain status of organisms and it was observed that chlorhexidine showed high bactericidal activity. The evidence presented here for the immediate and extended effect of 4 % chlorhexidine on large and sustained reductions in colonization by gram-negative and gram-positive organisms due to early application with chlorhexidine complements the Cochrane conclusion [14] that colonization is substantially lower among infants receiving topical antiseptics compared with those with no specific treatment (OR = 0.28; 95 % CI: 0.22–0.36).

There were few limitations to our study. The sample size limited our ability to definitively determine whether any of the study outcomes could be influenced by one intervention or the other. But the encouraging findings in our study were that most of the results were statistically significant. Our results provide evidence that the application of 4 % chlorhexidine solution on the umbilical cord provides protection to the baby from bacterial colonization and growth. The findings of our pilot study are important but need to be confirmed in a large community based trial with adequate statistical power as reduction in colonization and colony count in small number of subjects does not have adequate power to evaluate the impact on mortality or sepsis but does provide data on possible mechanisms.

The study cannot address an impact that these changes can have on the microbiome and its implications. From a comparison study in Pemba and Zambia (personal communication and in submission) it is clear that reduction in omphalitis may not essentially result in reduction in sepsis and mortality.

## Conclusions

Our study has shown that cord cleaning with the first time commercially produced 4 % chlorhexidine soon after birth can significantly reduce colonization as well as density of colonization among newborns. The study also demonstrated no impact of the control preparation compared to dry cord care on colonization (increase or decrease). The study showed impact of chlorhexidine on colonization in both hospital and community settings, but does not speak to relationship between this reduction and occurrence of sepsis or mortality. The findings of our pilot study are important but need to be tested in large community based trials to establish linkage between reduction in bacterial colonization and sepsis and or mortality. If the link between reduced colonization and reduction in neonatal mortality is established in larger trials in Africa, this intervention could have impact on neonatal mortality in developing country settings where there is limited availability of resources, stringent customs and poor environmental hygiene.

## Abbreviations

CI, confidence interval; GN, gram-negative; GP, gram-positive; OR, odds ratio; TBA, trained birth attendant

## Additional file

Additional file 1: Supplementary Tables (Supplementary Tables 1–3). (PDF 75 kb)

## References

[1] Lawn JE, Kinney MV, Black RE, Pitt C, Cousens S, Kerber K, Corbett E (2012). Health Policy Plan.

[2] Oestergaard MZ, Inoue M, Yoshida S, Mahanani WR, Gore FM, Cousens S (2011). Neonatal mortality levels for 193 countries in 2009 with trends since 1990: a systemic analysis of progress, projections and priorities. PLoS Med.

[3] UNICEF, WHO, The World Bank, the United Nations Population Division (2011). Levels and Trends in Child Mortality.

[4] Lawn JE, Cousens S, Zupan J, Lancet Neonatal Survival Steering Team (2005). 4 million Neonatal Deaths: When? Where? Why?. Lancet.

[5] Liu L, Johnson HL, Cousens S, Perin J, Scott S, Lawn JE (2012). Global, regional, and national causes of child mortality: an updated systematic analysis for 2010 with time trends since 2000. Lancet.

[6] Thaver D, Zaidi AKM (2009). Burden of neonatal infections in developing countries: a review of evidence from community-based studies. Pediatr Infect Dis J.

[7] Jellard J (1957). Umbilical cord as reservoir of infection in a maternity hospital. Br Med J.

[8] Remington JS, Remington JS, Klein JO (2001). Neonatal infection. Infectious Diseases of the Fetus and Newborn Infant.

[9] Goldenberg RL, McClure EM, Saleem S, Rouse D, Vermund S (2006). Use of vaginally administered chlorhexidine during labor to improve pregnancy outcomes. Obstet Gynecol.

[10] World Health Organisation (1998). Care of the Umbilical Cord: A review of the evidence.

[11] Mullany LC, Darmstadt GL, Katz J, Khatry SK, LeClerq SC, Adhikari RK (2006). Development of clinical sign based algorithms for community based assessment of omphalitis. Arch Dis Child Fetal Neonatal Ed.

[12] Mullany LC, Darmstadt GL, Katz J, Khatry SK, Leclerq SC, Adhikari RK (2009). Risk of mortality subsequent to umbilical cord infection among newborns of southern Nepal: cord infection and mortality. Pediatr Infect Dis J.

[13] Mullany LC, El Arifeen S, Winch PJ, Shah R, Mannan I, Rahman SM (2009). Impact of 4.0% chlorhexidine cleansing of the umbilical cord on mortality and omphalitis among newborns of Sylhet, Bangladesh: design of a community-based cluster randomized trial. BMC Pediatr.

[14] Zupan J, Garner P, Omari AA (2004). Topical umbilical cord care at birth. Cochrane Database Syst Rev.

[15] World Health Organization (2008). Review of the available evidence on 4% chlorhexidine solution for umbilical cord care. Second Meeting of the Subcommittee of the Expert Committee on the Selection and Use of Essential Medicines.

[16] Mullany LC, Darmstadt GL, Khatry SK, LeClerq SC, Katz J, Tielsch JM (2006). Impact of umbilical cord cleansing with 4.0% chlorhexidine on time to cord separation among newborns in southern Nepal: a cluster-randomized, community-based trial. Pediatrics.

[17] Larson EL and 1992, 1993, and 1994 APIC Guidelines Committee 18. APIC guideline for handwashing and hand antisepsis in health care settings. Source School of Nursing, Georgetown University, Washington, D.C., USA. [http://www.oc.lm.ehu.es/Fundamentos/fundamentos/LecturaDirigida/APIC%20hand_washing.pdf 1995] 10.1016/0196-6553(95)90070-5 7503437

[18] O’Grady NP, Alexander M, Dellinger EP, Gerberding JL, Heard SO, Maki DG (2002). Guidelines for the prevention of intravascular catheter related infections. The hospital Infection Control Practices Advisory committee, Center for Disease Control and Prevention, U.S. Pediatrics.

[19] McClure EM, Goldenberg RL, Brandes N, Darmstadt GL, Wright LL, CHX Working Group (2007). The use of chlorhexidine to reduce maternal and neonatal mortality and morbidity in low-resource settings. Int J Gynaecol Obstet.

[20] Mullany LC, Darmstadt GL, Khatry SK, Katz J, LeClerq SC, Shrestha S (2006). Topical applications of chlorhexidine to the umbilical cord for prevention of omphalitis and neonatal mortality in southern Nepal: a community-based, cluster-randomised trial. Lancet.

[21] Sinha A, Sazawal S, Pradhan A, Ramji S, Opiyo N (2015). Chlorhexidine skin or cord care for prevention of mortality and infections in neonates. Cochrane Database Syst Rev.

[22] Seeberg S, Brinkhoff B, John E, Kjellmer I (1984). Prevention and control of neonatal pyoderma with chlorhexidine. Acta Paediatr Scand.

[23] Parashar UD, Bennett JV, Boring JR, Hlady WG (1998). Topical antimicrobials applied to the umbilical cord stump: a new intervention against neonatal tetanus. Int J Epidemiol.

[24] Mullany LC, Darmstadt GL, Katz J, Khatry SK, LeClerq SC, Adhikari RK (2007). Risk factors for umbilicalCord infection among newborns of southern Nepal. Am J Epidemiol.

[25] Mullany LC, Saha SK, Shah R, Islam MS, Rahman M, Islam M, Talukder RR (2012). Impact of 4.0% chlorhexidine cord cleansing on the bacteriologic profile of the newborn umbilical stump in rural Sylhet District, Bangladesh: a community-based, cluster-randomized trial. Pediatr Infect Dis J.

[26] Hodgins S, Thapa K, Khanal L, Aryal S, Suvedi BK, Baidya U (2010). Chlorhexidine gel versus aqueous for preventive use on umbilical stump: a randomized noninferiority trial. Pediatr Infect Dis J.

[27] James L, Hoppe-Bauer JE, Isenberg HD (1992). Processing and interpretation of lower respiratory tract specimen. Clinical microbiology procedures handbook.

[28] Goldenberg RL, McClure EM, Saleem S (2013). A review of studies with chlorhexidine applied directly to the umbilical cord. Am J Perinatol.

[29] Arifeen SE, Mullany LC, Shah R, Mannan I, Rahman SM, Talukder MR (2012). The effect of cord cleansing with chlorhexidine on neonatal mortality in rural Bangladesh: a community-based, cluster-randomised trial. Lancet.

[30] Belfrage E, Enocksson E, Kalin M, Marland M (1985). Comparative efficiency of chlorhexidine and ethanol in umbilical cord care. Scand J Infect Dis.

[31] Athanassiadis B, Abbott PV, Walsh LJ (2007). The use of calcium hydroxide, antibiotics and biocides as antimicrobial medicaments in endodontics. Aust Dent J.

[32] Gomes BP, Souza SF, Ferraz CC, Teixeira FB, Zaia AA, Valdrighi L (2003). Effectiveness of 2% chlorhexidine gel and calcium hydroxide against Enterococcus faecalis in bovine root dentine in vitro. Int Endod J.

[33] Gomes BP, Sato E, Ferraz CC, Teixeira FB, Zaia AA, Souza- Filho FJ (2003). Evaluation of time required for recontamination of coronally sealed canals medicated with calcium hydroxide and chlorhexidine. Int Endod J.

[34] Mohammadi Z, Abbott PV (2009). The properties and applications of chlorhexidine in endodontics. Int Endod J.

[35] Darmstadt GL, Bhutta ZA, Cousens S, Adam T, Walker N, de Bernis L (2005). Evidence based cost effective interventions: how many newborn babies can we save?. Lancet.

[36] Mullany LC, Khatry SK, Sherchand JB, LeClerq SC, Darmstadt GL, Katz J (2008). A randomized controlled trial of the impact of chlorhexidine skin cleansing on bacterial colonization of hospital-born infants in Nepal. Pediatr Infect Dis J.

