# Supplementary material for: Effect of 4 % chlorhexidine on cord colonization among hospital and community births in India: a randomized controlled study
Source: BMC Pediatr. 2016 Aug 2;16:121. doi: 10.1186/s12887-016-0625-7 (PMC4971644; doi:10.1186/s12887-016-0625-7)
Supplement: Additional file 1: — Supplementary Tables (Supplementary Tables 1–3). (PDF 75 kb) [file 12887_2016_625_MOESM1_ESM.pdf]

Supplementary Tables

Supplementary Table 1 Bacterial colonization by intervention group, time of swab collection and strain in hospital births (limited to samples with growth)

Variables	Chlorhexidine (n=86)			Placebo (n=86)			Dry cord care (n=75)		
	GP*	GN#	Both†	GP*	GN#	Both†	GP*	GN#	Both†
0 hours	10 (11.6)	13 (15.1)	0 (0.0)	4 (4.7)	9 (10.5)	0 (0.0)	5 (6.7)	7 (9.3)	2 (2.7)
2 hours post intervention	6 (6.9)	2 (2.3)	0 (0.0)	14 (16.3)	8 (9.3)	1 (1.2)	12 (16.0)	8 (10.7)	4 (5.3)
48 hours post intervention	8 (9.3)	2 (2.3)	2 (2.3)	22 (25.6)	19 (22.1)	17 (22.7)	29 (38.7)	14 (18.7)	10 (13.3)

*Gram positive

Gram negative

†Both gram positive and gram positive

Supplementary Table 2 Bacterial colonization by intervention group, time of swab collection and strain among community births (limited to samples with growth)

	Chlorhexidine (n=36)			Placebo (n=24)			Dry Cord care (n=19)		
**Variables**	**GP***	**GN#**	**Both†**	**GP***	**GN#**	**Both†**	**GP***	**GN#**	**Both†**
0 hr	3 (8.3)	12 (33.3)	15 (41.7)	5 (20.8)	8 (33.3)	7 (29.2)	2 (10.5)	4 (21.1)	8 (42.1)
2 hrs post intervention	9 (25.0)	4 (11.1)	3 (8.3)	13 (54.2)	2 (8.3)	0 (0.0)	2 (10.5)	5 (26.3)	7 (36.8)
48 hrs post intervention	4 (11.1)	7 (19.4)	3 (8.3)	5 (20.8)	3 (12.5)	8 (33.3)	5 (26.3)	3 (15.8)	8 (42.1)

*Gram positive

Gram negative

†Both gram positive and gram positive

Supplementary Table 3 Comparison between chlorhexidine vs. placebo/dry cord care for the bacterial colonization stratified by gram positive/gram negative organisms

Variables	*Chlorhexidine vs. Placebo*		*Chlorhexidine vs. Dry cord*	
	Odds ratio(95%CI)	*p value*	*Odds ratio(95%CI)*	*p value*
HOSPITAL				
**Gram positive: Paired comparison for bacterial colonization between-**				
Baseline and 2-hour post intervention	0.27 (0.09-0.83)	0.02	0.22 (0.07-0.63)	0.005
Baseline and 48-hour post intervention	0.16 (0.07- 0.35)	0.00	0.12 (0.05-0.27)	<0.001
**Gram negative: Paired comparison for bacterial colonization between-**				
Baseline and 2-hour post intervention	0.15 (0.03-0.80)	0.03	0.13 (0.03-0.63)	0.01
Baseline and 48-hour post intervention	0.07 (0.02- 0.21)	0.00	0.11 (0.04-0.34)	<0.001
COMMUNITY				
**Gram positive: Paired comparison for bacterial colonization between-**				
Baseline and 2-hour post intervention	0.36 (0.11-1.17)	0.09	0.50 (0.13-1.84)	0.30
Baseline and 48-hour post intervention	0.17 (0.05-0.59)	0.006	0.10 (0.03-0.38)	0.001
**Gram negative: Paired comparison for bacterial colonization between-**				
Baseline and 2-hour post intervention	2.39 (0.44-12.93)	0.31	0.06 (0.01-0.33)	0.001
Baseline and 48-hour post intervention	0.42 (0.14-1.27)	0.12	0.23 (0.07-0.79)	0.02
